# Virtual reality method to analyze visual recognition in mice

**DOI:** 10.1371/journal.pone.0196563

**Published:** 2018-05-16

**Authors:** Brent Kevin Young, Jayden Nicole Brennan, Ping Wang, Ning Tian

**Affiliations:** Department of Ophthalmology and Visual Sciences, John Moran Eye Center, University of Utah, School of Medicine, Salt Lake City, Utah, United States of America; Tokai University, JAPAN

## Abstract

Behavioral tests have been extensively used to measure the visual function of mice. To determine how precisely mice perceive certain visual cues, it is necessary to have a quantifiable measurement of their behavioral responses. Recently, virtual reality tests have been utilized for a variety of purposes, from analyzing hippocampal cell functionality to identifying visual acuity. Despite the widespread use of these tests, the training requirement for the recognition of a variety of different visual targets, and the performance of the behavioral tests has not been thoroughly characterized. We have developed a virtual reality behavior testing approach that can essay a variety of different aspects of visual perception, including color/luminance and motion detection. When tested for the ability to detect a color/luminance target or a moving target, mice were able to discern the designated target after 9 days of continuous training. However, the quality of their performance is significantly affected by the complexity of the visual target, and their ability to navigate on a spherical treadmill. Importantly, mice retained memory of their visual recognition for at least three weeks after the end of their behavioral training.

## Introduction

Behavioral tests have been extensively used to study the visual recognition of animals. Recent developments in virtual reality (VR) tests of animal behavior has provided more consistent testing conditions, less human-animal interaction, opportunities to use a wider variety of experimental parameters, and more precise measurements of the perception of the tested animals. VR tests have been applied across a wide variety of disciplines from disease treatment in humans, to study of navigation and memory in primates, rodents, and even insects [1–9]. Despite the fact that many VR tasks use visual cues, most of the experiments in rodents which use VR probe the function of the hippocampus or memory formation/retrieval but not visual perception [10–17]. Currently, the most commonly used behavior test for visual function is the optokinetic test [18–21]. However, this test does not measure visual perception, but a reflex response. In addition, the optokinetic test is limited to testing spatial frequency or contrast sensitivity to horizontally drifting vertical bars [20]. Many fundamental questions, such as how quickly an experimental animal can learn to recognize certain visual targets, how significantly the complexity of visual cues affects the training requirements and performances of animals, and how long the visual memory will last once animals are trained, remain unaddressed.

In this study, we developed a VR behavior test to characterize fundamental features of training mice for visual perception. We show that mice were able to recognize the designated visual targets after 9 days of training. However, their performance is significantly affected by the complexity of the visual targets. In addition, mice retained a memory of their visual recognition following a break in their behavioral training.

## Materials and methods

### Animals

All animals used for this study were wild type C57BL/6 mice aged P25-P80. During the training period, mice were on a restricted diet and the body weight was monitored daily (see details below). All animal procedures and care were performed following protocols approved by the IACUC of the University of Utah in compliance with PHS guidelines and with those prescribed by the Association for Research in Vision and Ophthalmology (ARVO).

### VR test system

**Fig 1:**
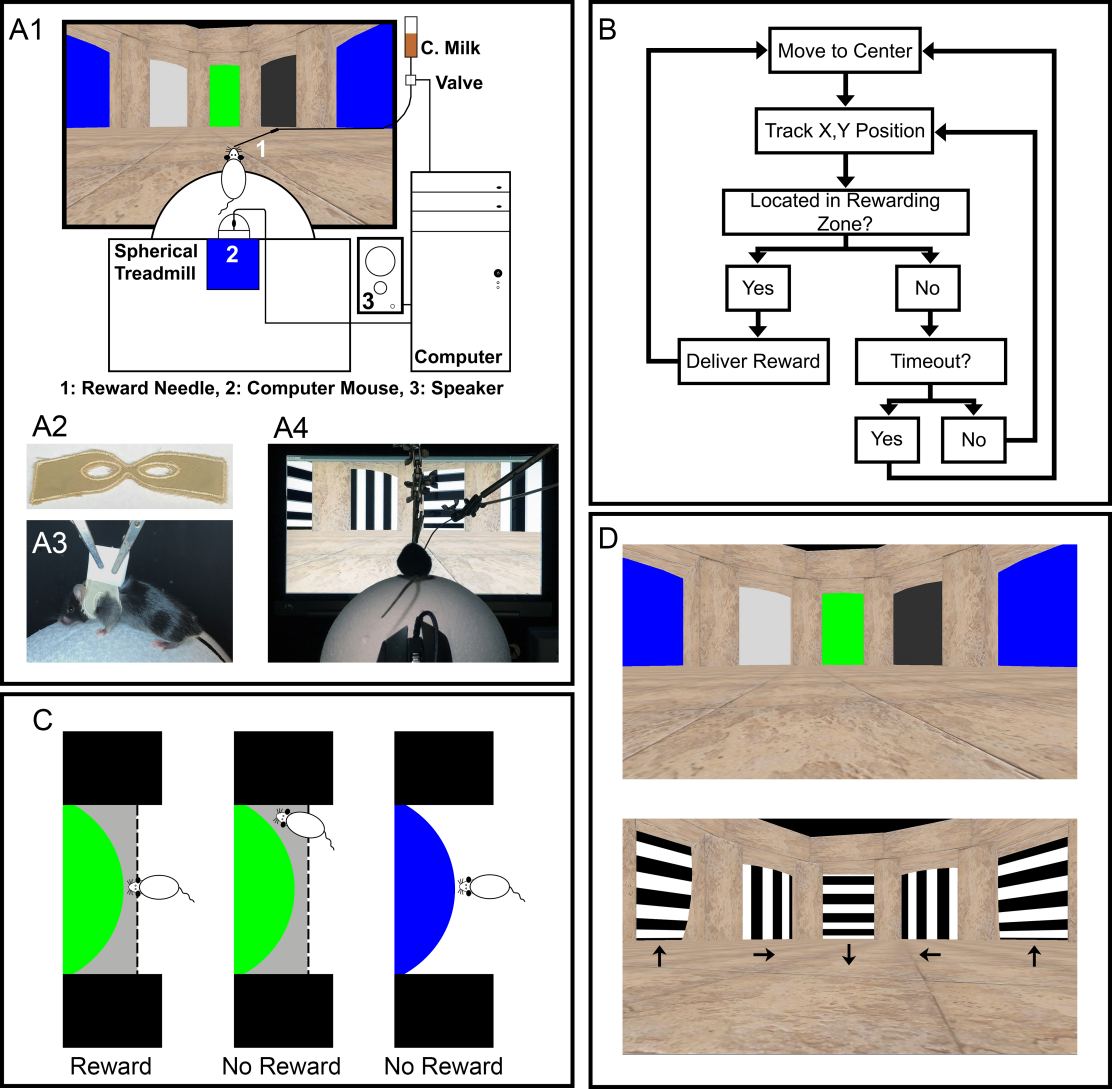
Outline of VR behavior test setup. **A**) Overview of the equipment setup for the VR experiment (A1), the harness used to hold the mouse (A2), a mouse in a harness on the spherical treadmill (A3), and an image of a mouse performing in the VR environment (A4). **B**) Logic diagram of the VR program. **C**) Illustration of the conditions required for a mouse to receive a reward at the designated target (green). The shaded area represents where the mouse must be when facing the target to receive a reward. **D**) A view of both the colored/luminance targets (top), and the moving bars (bottom). Arrows are added to the moving bars to indicate the direction of movement.

The VR system was constructed in a similar manner to the one described previously [5]. Briefly, the system consists of a control computer (HP Pavilion HPE, HP Inc., Palo Alto, CA, USA) with dual displays, which runs a custom-written VR program. One monitor displays the visual targets to the mouse, while the other monitor displays the visual targets as well as other control parameters to the experimenter. The size of the monitor for mouse is 51.28 cm wide and the distance to the mouse from the monitor is 46 cm, which results in a field of view angle of 58.3°. A spherical treadmill [22] is coupled to a high performance computer mouse (Logitech, G502, Silicon Valley, CA, USA), which feeds the movement of the spherical treadmill into the VR program. The VR program also drives a rewarding system to deliver a small amount of chocolate milk to the mouse when the mouse correctly identifies the rewarding target. An IR-sensitive video camera was used to monitor the performance of the mouse. Additionally, an audio cue was used to indicate reward delivery during the training to facilitate learning. The spherical treadmill, the monitor for displaying visual targets, the chocolate milk feeding needle, the speaker for providing audio cue, and the video camera are placed inside a light-tight box to reduce experimental disruptions (Fig 1, part A1).

### Behavioral training and testing

Prior to any training, all mice were individually caged, and given access to a running wheel in their home cage. Mice were then food restricted, and monitored daily. Food was given each day to maintain a body weight that was 85–90% of starting weight. After two weeks of food restriction, each mouse was dressed into a custom-made harnesses (Fig 1, part A2) under anesthesia of 2–3% isoflurane gas using a Vapomatic anesthesia system (Bickford Inc., Wales Center, NY, USA). A harness was affixed to itself in the back using staples, and then wrapped with tape to prevent harm to the mouse (Fig 1, part A3). Harnesses remained on the mice during the course of the training and testing. The following day mice were introduced to the VR set up by clipping their harnesses above the spherical treadmill (Fig 1, part A3). Training sessions occurred for each mouse twice per day, and each session was 30 minutes. Harnesses were removed at the end of training/testing under anesthesia of 2–3% isoflurane.

The VR program was custom written using the Unity game engine (Unity Technology, https://unity3d.com/unity). The logic diagram of the program is outlined in Fig 1B. Briefly, the tested mouse was virtually placed at the center of a circular arena (2.4 meters in diameter) with a random orientation. Its X, Y position in the arena was continuously tracked using the input from the computer mouse. The tested mouse was then trained to navigate to a designated “Reward” target through moving the Styrofoam ball of the spherical treadmill. Once it properly arrived at the reward target and faced the center of the target for one second (Fig 1C), an audio cue played and a small amount (~100 μL) of chocolate milk was delivered through a feeding needle. The display of the arena was then automatically readjusted so that the tested mouse was placed back at the center of the arena (see S1 Video for a representative performance). If the tested mouse was unable to reach the reward target in 220 seconds, there would be a time out “Fail,” and the display was reset so that the tested mouse was replaced back at the center of the arena. The same procedure continued for 30 minutes, which constituted one “Session.”

We used two different sets of visual targets for this study. One set consisted of uniform areas in the following solid colors: green, blue, light grey, and dark grey. These are hereafter referred to as the “colored” targets. The brightness of these objects was measured 46 cm away from the screen using an optical power meter (Model 371, UDT Instruments, Baltimore, MD, USA), and are as follows: green, 138.50 nW; blue, 87.31 nW; light grey, 224.80 nW; and dark grey, 7.37 nW. The second set of targets consisted of black and white bars moving in four different directions: down, up, left, and right. These are referred to as “moving bars” (Fig 1D, bottom panel). The width of the black and white bars (15.88 mm) was calibrated, so that when the mice were the farthest distance away (0.44 cycles/degree), or the closest (0.04 cycles/degree) it was within the known visible spatial frequency for C57BL/6 mice [18,19].

**Fig 2:**
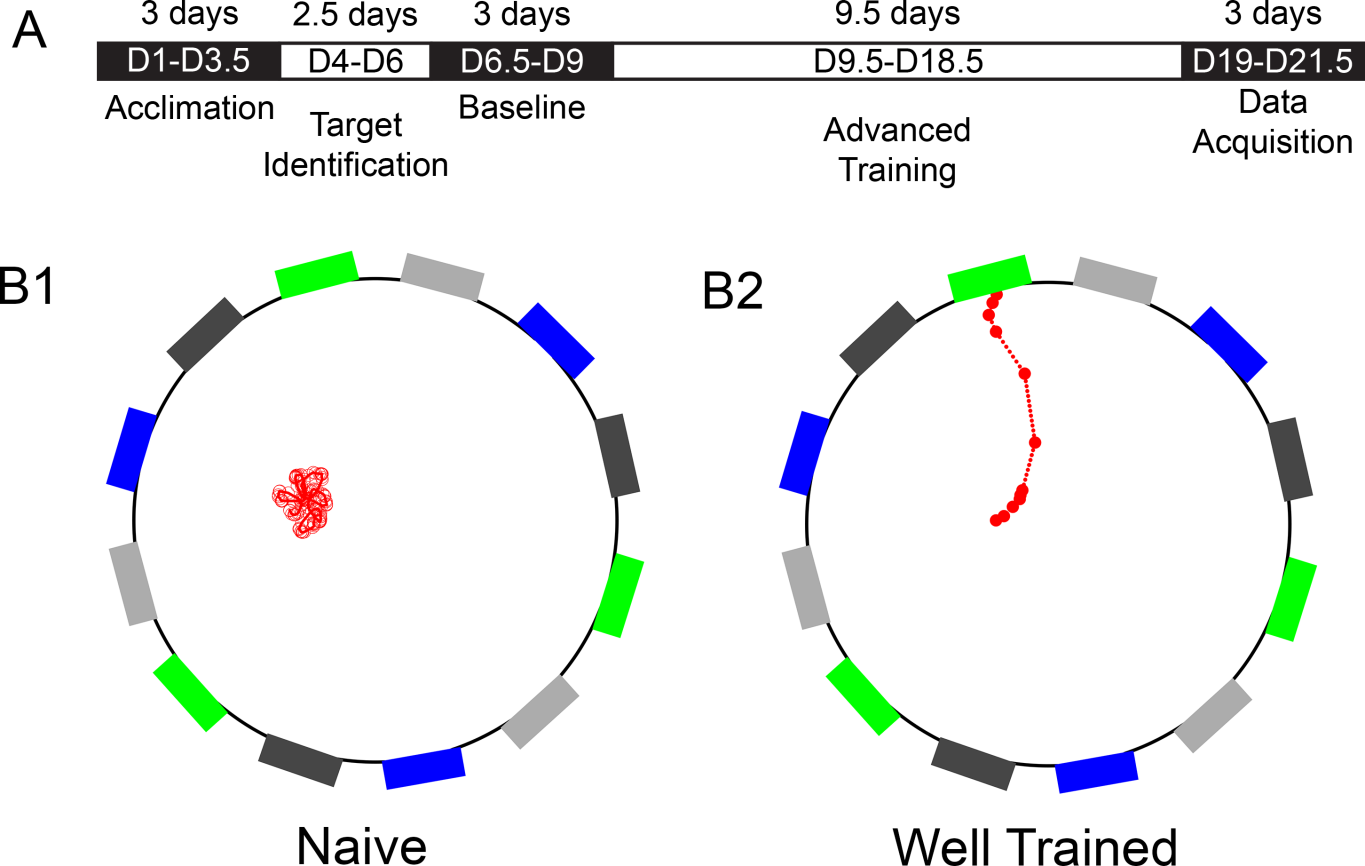
Training outline and representative tracing results. **A**) An outline of the training schedule for the color/luminance recognition. **B**) Representative tracings (red dots and lines) of the movement of the mouse in the VR arena (black circle). The color-coded rectangles around the circle indicate the visual targets. B1 shows a representative tracing of a mouse that has just started training. B2 shows a representative tracing of a mouse that has been trained for 21 days.

Fig 2A shows the training/testing schedule for colored target and moving bar recognition. The entire training/testing procedure consisted of five phases. During the first phase (Acclimation), no visual display was provided from the target monitor. Mice were trained to maneuver on the spherical treadmill, acclimated to drinking from the feeding needle, and were rewarded with chocolate milk for moving forward on the spherical treadmill. During the second phase (Target Identification), the VR display was turned on, and the experimenter manually rotated the ball of the spherical treadmill to guide the mouse to the desired rewarding target. These two phases were used only for training, without data collection. However, to determine how naïve mice perform during the Acclimation phase, 5 mice were recorded during the Acclimation phase without any training for target identification. Data from these mice is used as a reference of untrained mice at the D1-D3.5 time points. After the initial two phases of training (Acclimation and Target Identification), the mice were allowed to navigate the arena without any human interference. For the next phase (Baseline), the performance of the mice was recorded as a baseline. Mice that did not drink chocolate milk from the feeding needle, or those unable to arrive at a rewarding target at least 6 times during a single session (30 minutes) were removed from further training and testing. About 50% of the mice were removed at this point, and they were not included in the final data analysis.

The remaining mice received additional training every day for 9.5 days (Advanced Training). The final measurement of their performance occurred during the last phase (Data Acquisition). To test the behavioral memory, three mice were given a break for 22 days after their training and testing. During this break harnesses were removed and the mice were allowed free access to food. Prior to memory recall testing, the harness was once again placed on the mice and food restriction was reinitiated. Mice were then placed back on the VR setup with no additional training, and allowed to locate the same reward target as before.

### Statistical analysis

Data is all presented as mean ± SEM in the text and figures (Igor Pro, WaveMetrics, Inc., Lake Oswego, OR). Student t-tests, Analysis of Variance (ANOVA), and Kolmogorov-Smirnov test (K-S) tests were used to examine the difference between means and distributions using Statview (Abacus Concepts, Berkeley, CA, USA). Fisher’s protected least significant difference (Fisher’s PLSD) tests were used for pairwise comparison of groups following an ANOVA test. Chi-squared tests were performed using equations coded in Excel (Microsoft Crop., Redmond, WA, USA). The level of statistical significance was set at 5%. In all figures, * indicates P<0.05; ** indicates P<0.01; and *** indicates P<0.001.

## Results

### Mice can be trained for visual recognition in 9 days

**Fig 3:**
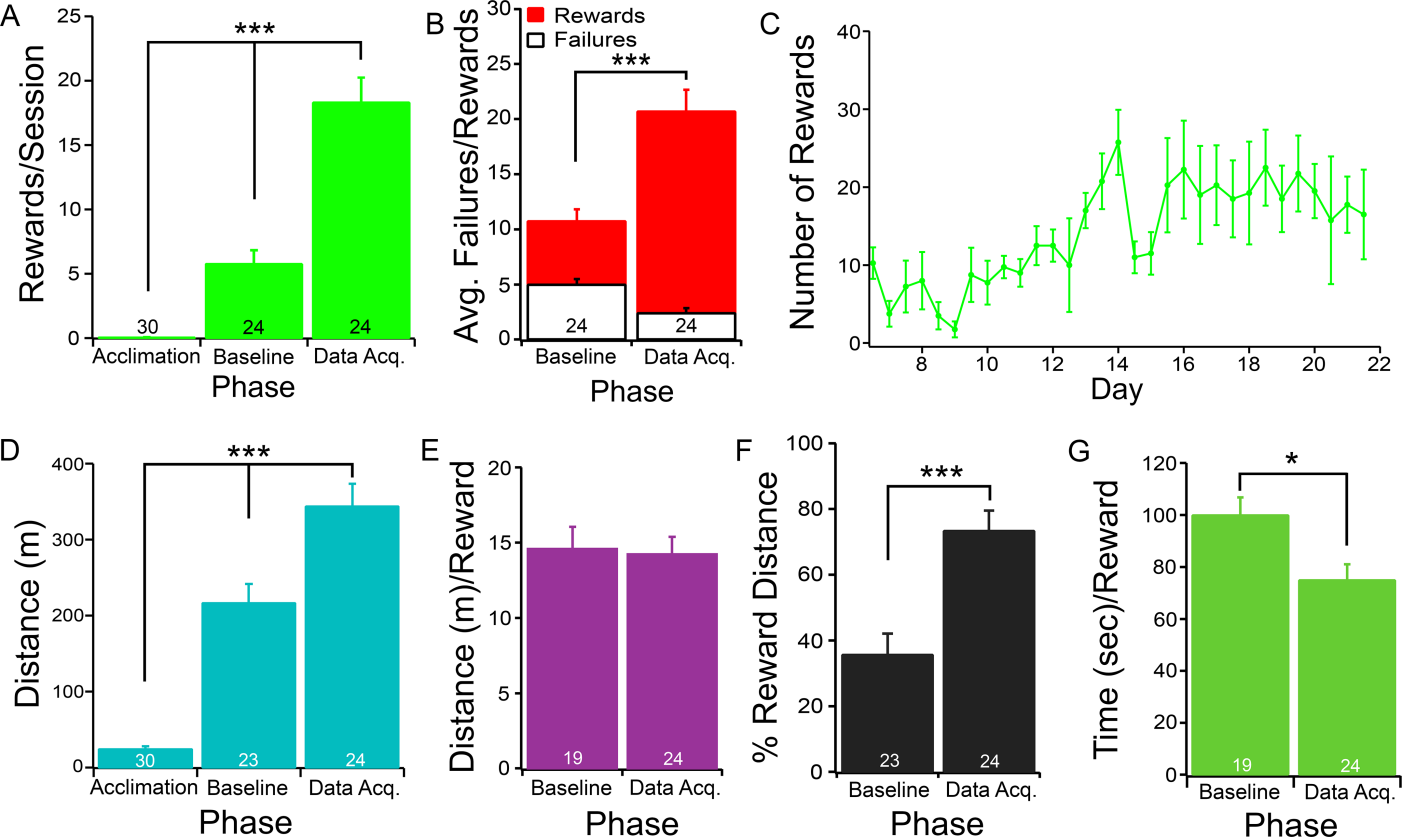
The time course of the recognition of color/luminance targets. The average number of rewards per session, the average distance traveled to reach a reward, and the average time required to reach a reward are analyzed as a function of training time. **A**) The average number of rewards per session of untrained mice (Acclimation, D1-D3.5), mice after Baseline training (Baseline, D6.5-D9), and mice at the end of training (Data Acq, D19-D21.5). An ANOVA test shows that the differences among these three groups are statistically significant (F(2,75) = 62.18, P<0.001). Fisher’s PLSD post-hoc analysis shows that the differences between all three pair-wise comparisons are statistically significant (Acclimation versus Baseline, P<0.001; Acclimation versus Data Acq., P<0.001; Baseline versus Data Acq., P<0.001). **B**) The average number of rewards and failures during the Baseline and Data Acquisition phases. A Chi-squared test shows that the difference between these two groups is statistically significant (F = 114, P<0.001). **C**) The average number of rewards per session as a function of training time. **D**) The total distance traveled per session during the Acclimation, Baseline, and Data Acquisition phases. An ANOVA test shows that the differences among these three groups are statistically significant (F(2,75) = 63.64, P<0.001). Fisher’s PLSD post-hoc analysis shows that the differences between all three pair-wise comparisons are statistically significant (Acclimation versus Baseline, P<0.001; Acclimation versus Data Acq., P<0.001; Baseline versus Data Acq., P<0.001). **E**) The average distance traveled per reward during the Baseline and Data Acquisition phases. A paired Student t-test shows that the difference between these two groups is not statistically significant (df = 18, t = 0.02, P>0.05). **F**) The percent of the total distance traveled that resulted in rewards during the Baseline and Data Acquisition phases. A paired Student t-test shows that the difference between these two groups is statistically significant (df = 22, t = 4.69, P<0.001). **G**) The average time required for reaching a reward per session during the Baseline and Data Acquisition phases. A paired Student t-test shows that the difference between these two groups is statistically significant (df = 18, t = 2.24, P<0.05). In all panels except panel C, the number in each column indicates the number of test sessions of 4 mice. Data are presented in the bar graphs as mean ± SEM in this figure and all of the following figures.

We first examined how long it would take to train mice to perform a VR test to distinguish cues different in color and luminance. To determine if the mice can correctly recognize the designated color/luminance target, their movements were traced to quantify how often they arrived at each target during each session. When mice were initially introduced to the VR setup they tend to hold still, not moving enough to reach the periphery of the arena where the targets lie (Fig 2, part B1). At the end of the training/testing (phase 5), all mice wander freely around the arena looking for rewarding targets, even able to go directly to the rewarding targets from the center of the arena (Fig 2, part B2). After the first 9 days of training (Acclimation, Target Identification, and Baseline), mice can regularly arrive at the correct target (Fig 3A).

### Mice improve their performance after initial training

Despite the mice being able to recognize the designated rewarding target within 9 days of training, the number of successful arrivals at the designated rewarding target in each session continues to increase with further training (Fig 3A–3C). Fig 3C plots the average number of successful arrivals at the designated target per session as a function of training time and shows that the number of rewards per session reaches the peak at day 13–14 of training, and then plateaus afterwards. Quantitatively, the average number of successful arrivals at the designated rewarding target per session was 0.03±0.03 for naïve mice during the Acclimation phase (D1-D3.5), 5.75±1.07 during the Baseline phase (D6.5-D9), and 18.29±1.95 during the Data Acquisition phase. An ANOVA test shows that the differences among these three groups are statistically significant (Fig 3A, F(2,75) = 62.18, P<0.001). Fisher’s PLSD post-hoc analysis shows that the differences between all three paired comparisons are statistically significant (Acclimation versus Baseline, P<0.001; Acclimation versus Data Acq., P<0.001; Baseline versus Data Acq., P<0.001). A Chi-squared test shows that the increase in the ratio of rewards versus failures significantly improved by the end of the Data Acquisition phase (Fig 3B, 1.5:1 at Baseline phase, to 7.6:1 at Data Acquisition phase, F = 114, P<0.001).

To determine how mice improve their performance with training, we analyzed their movement in more detail. Our results show that, after the advanced training, mice traveled a farther distance during each session (Fig 3D) without reducing the distance for each reward (Fig 3E). The average distance per session was 24.39±3.49 meters (m) during the Acclimation phase. During the Baseline phase it was 216.81±24.98 m/session, and finally 343.80±29.75 m/session during the Data Acquisition phase (Fig 3D). An ANOVA test shows that the differences among these three groups are statistically significant (F(2,75) = 63.64, P<0.001). Fisher’s PLSD post-hoc analysis shows that the differences between all three pair-wise comparisons are statistically significant (Acclimation versus Baseline, P<0.001; Acclimation versus Data Acq., P<0.001; Baseline versus Data Acq., P<0.001). On the other hand, the average distance traveled for each reward was 14.59±1.45 m during the Baseline phase, and 14.24±1.14 m during the Data Acquisition phase. A paired Student t-test shows that this reduction is not statistically significant (Fig 3E, df = 18, t = 0.02, P>0.05).

To determine if the mice spent a greater percentage of running distance arriving at rewarding targets after advanced training, we computed the percent of total distance that was used for receiving rewards during different phases. This distance was increased from 35.7±6% during the Baseline phase to 73.4±6% during the Data Acquisition phase (Fig 3F). A paired Student t-test shows that the difference between these two groups is statistically significant (df = 22, t = 4.69, P<0.001). The increased total running distance per session was accompanied by a reduced time required to arrive at each reward (Fig 3G). In average, mice spent 100.00±6.8 seconds (sec) to reach a reward during the Baseline phase. However, the time required for each reward was decreased to 75.07±5.9 sec per reward during the Data Acquisition phase (Fig 3G). A paired Student t-test shows that the difference between these two groups is statistically significant (df = 18, t = 2.24, P<0.05). All together, these results suggest that the training of the VR behavioral test seems to have two components, the training for visual recognition, and the training for maneuvering the treadmill. The training for the visual recognition is fast (9 days), but the training for controlling the treadmill requires more time and effort.

### Mice are able to distinguish bars moving in different directions

**Fig 4:**
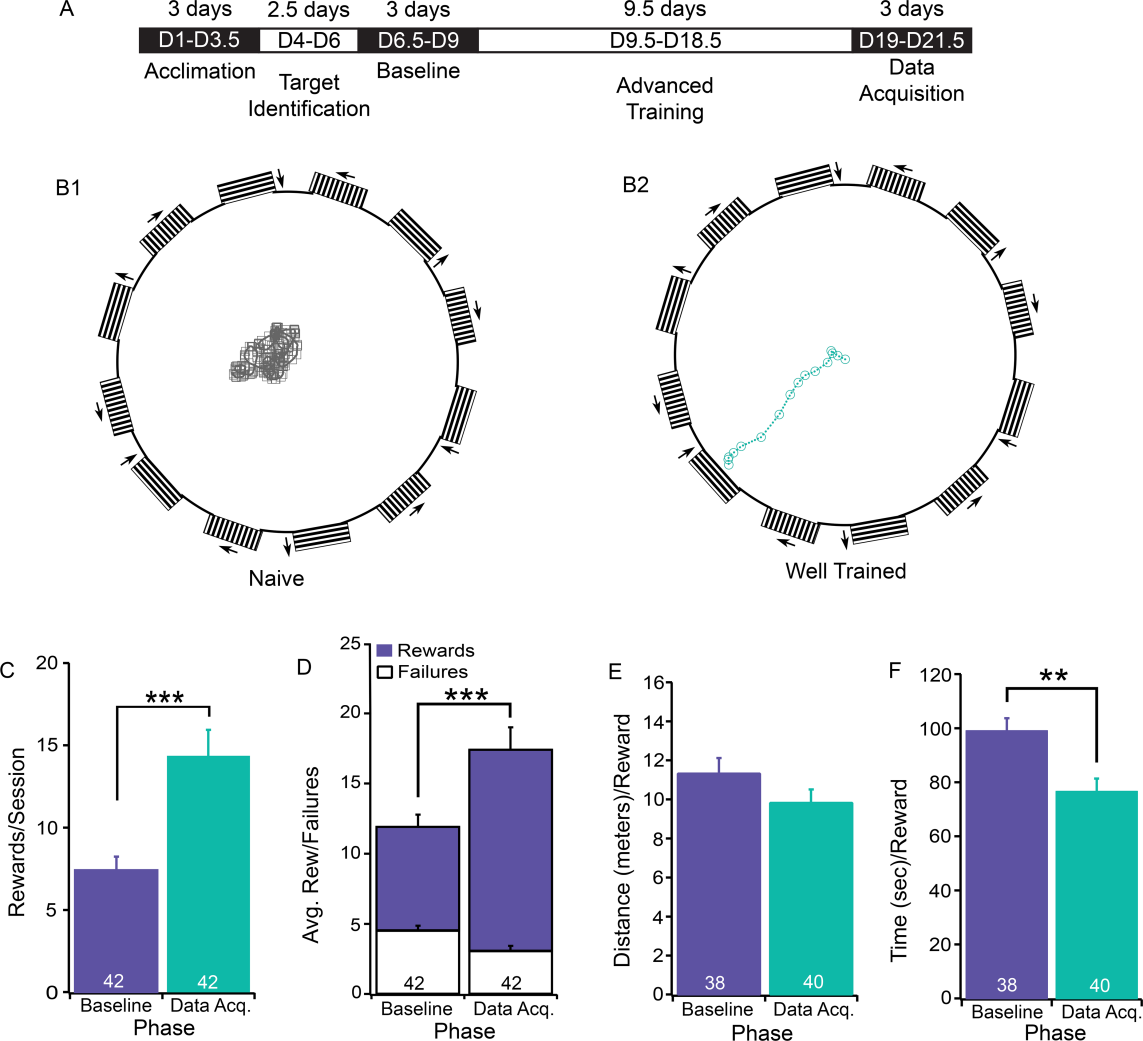
The time course of the recognition of moving bars. **A**) The outline of the training schedule for the recognition of moving bars. **B**) Representative tracing results of a mouse that has just started training (Acclimation, B1), and a mouse that has been trained to recognize the downward moving target for 21 total days (Data Acquisition, B2). **C**) The average number of rewards per session during the Baseline phase (day 6.5–9), and the Data Acquisition phase (day 19–21.5). A paired Student t-test shows that the difference between these two groups is statistically significant (df = 41, t = 3.76, P<0.001). **D**) The average number of rewards and failures during the Baseline and Data Acquisition phases. A Chi-squared test shows that the difference between these two groups is statistically significant (F = 63.9, P<0.001). **E**) The average distance traveled for each reward during the same two phases as in Panel C. A paired Student t-test shows that the difference between these two groups is not statistically significant (df = 37, t = 1.28, P>0.05). **F**) The average time required for each reward during the same two phases as Panel C. A paired Student t-test shows that the difference between these two groups is statistically significant (df = 37, t = 3.13, P<0.01). The number in each column indicates the number of test sessions from 7 mice.

We then examined whether mice can distinguish more complex visual targets and whether they require more training for more complex visual targets. Accordingly, we trained a group of mice to distinguish a set of black/white bars moving toward four different directions (Fig 1D, bottom). Similar to the color/luminance recognition, mice are able to distinguish targets moving in different directions after training. Fig 4B shows representative tracing results of a mouse that has just started training during the Acclimation phase (B1) and a mouse that was at the Data Acquisition phase (B2). After initial training, the mice were able to correctly distinguish the rewarding downward moving target from targets moving in the other three directions. Mice increased the number of times they reached the rewarding target from 7.43±0.87/session during the Baseline phase to 14.36±1.58/session during the Data Acquisition phase. A paired Student t-test shows that the difference between these two groups is statistically significant (Fig 4C, df = 41, t = 3.76, P<0.001). In addition, a chi-squared test was used to examine the difference in the ratio of rewards versus failures between these two groups and showed that the ratio of rewards is significantly higher in the Data Acquisition phase (4.67:1) than the Baseline phase (1.63:1, Fig 4D; F = 63.9, P<0.001).

Similar to color/luminance recognition, mice trained to distinguish the moving direction of moving bars also improved their performance over the course of training. The mice reduced the average time required for reaching each reward from 98.93±4.7 sec in the Baseline phase to 78.40±4.5 sec in the Data Acquisition phase. A paired Student t-test shows that the difference between these two groups is statistically significant (df = 37, t = 3.13, P<0.01) (Fig 4F). However, the average distance required reaching each reward did not change significantly (Fig 4E, paired Student t-test, df = 37 t = 1.28, P>0.05).

### Mice retain memory of their virtual reality perception

**Fig 5:**
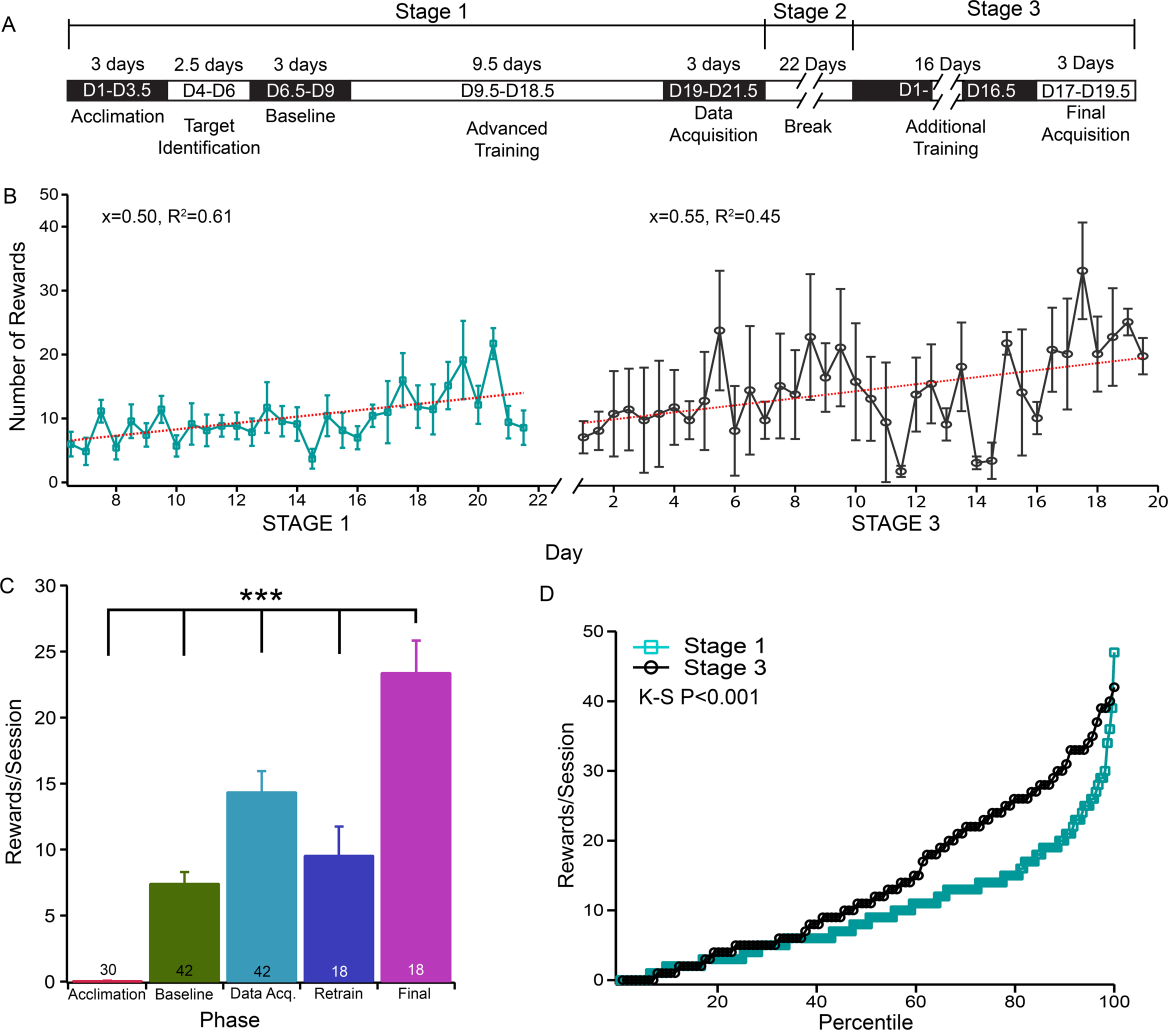
Mice retain memory of their VR perception. **A**) The outline of the training/testing schedule of the memory test of VR recognition. **B**) The average number of rewards per session as a function of training time during stage 1 (left, teal) and stage 3 (right, black). A linear regression analysis shows a linear fitting for both phases with a R^2^ = 0.61 and 0.45, respectively. **C**) The number of rewards per session of the Acclamation phase (Stage 1), Baseline phase (Stage 1), Data Acquisition phase (Stage 1), the first six sessions of Stage 3 of Additional Training (Retrain), and the final six sessions of Stage 3 Final Acquisition (Final). An ANOVA test shows that the differences among these five groups are statistically significant (F(4,145) = 29.66, P<0.001). Fisher’s PLSD post-hoc analysis shows that the number of rewards of the final six sessions (Final) is significantly higher than any other training phase. In addition, the number of rewards of the initial six sessions of the third stage (Retrain phase) is statistically higher than that of the Acclimation phase of the first stage (Fisher’s PLSD, P<0.0001) but is not statistically different from that of the Baseline (Fisher’s PLSD, p>0.05). However, it is statistically different from that of the Data Acquisition phases of the first stage (Fisher’s PLSD, P<0.05). The number in each column indicates the number of test sessions for 7 mice during stage 1, and 3 mice during stage 3. **D**) Cumulative distributions of the frequency of rewards/session in stage 1 and stage 3. A Kolmogorov-Smirnov (K-S) test confirmed that the difference of the distributions of the rewards/session of these two stages is statistically significant (P<0.001).

Finally, we examined whether mice could retain the capability of visual recognition and VR performance after they were trained. Accordingly, we established a 3-stage training/testing protocol (Fig 5A). During the first stage, a group of mice were trained for 21 days to recognize a downward moving target following the same schedule illustrated in Fig 2A. As shown in Fig 5B, the number of rewards per session increased after this training period, but did not reach a plateau. During the second stage, the mice were held in their home cages without any training and given free food access for 22 days. During the third stage, the mice were reintroduced to VR training for an additional 16 days and then we evaluated their performance. When the mice were reintroduced to training during the third stage, there was no Acclimation, or Target Identification phase (Fig 5A). To determine how well the memory was retained over the three-week break period (the second stage), the number of rewards per session during the first six sessions of the third stage (9.56±2.2) was compared to that of the Acclimation phase (day 1–3.5; 0.03±0.03 rewards/session), Baseline phase (day 6.5–9; 7.43±0.87 rewards/session), the initial Data Acquisition phase (day 19–21.5; 14.36±1.6 rewards/session) of the first stage, and the final six sessions of stage 3 (Final phase, 23.39±2.4 rewards/session). The average number of rewards during the initial six sessions of the third stage (Retrain phase) is statistically higher than that of the Acclimation phase of the first stage (Fisher’s PLSD, P<0.0001), and the average number of rewards during the final six sessions (Final) is significantly higher than any other training phase. However, the average number of rewards during the first six sessions of the third stage (Retrain phase) is not statistically different from that of the Baseline (Fisher’s PLSD, p>0.05) but is statistically different from that of the Data Acquisition phases of the first stage (Fisher’s PLSD, P<0.05) (see S1 Table for detailed results of Fisher’s PLSD tests). Fig 5D shows the cumulative distributions of the frequency of rewards/session of stage 1 and stage 3 of training. It is evident that the mice had a more rapid increase in rewards per session during stage 3, than they did during stage 1. A Kolmogorov-Smirnov (K-S) test confirmed that the difference of the distributions of the rewards/session of these two stages is statistically significant (P<0.001). Therefore, the non-maximum training during stage 1 facilitates the learning during stage 3.

## Discussion

In this study, we report a computerized and quantitative VR behavioral test to evaluate visual perception of mice. We emphasized the time requirement for the recognition of visual targets with different complexity. In comparison with commonly used visual behavioral tests, such as the lever press test [23–25], Morris water maze [26–28] and OKR [19–21], the VR test provides several advantages. First, this test can use virtually any type of visual targets, from simple displays such as color, luminance and moving targets, to complex visual displays such as a natural scene. Therefore, this test is readily applicable for use to analyze complex visual questions. Although the mice are able to quickly learn the task and differentiate the target of choice, the type of visual target makes a difference, with the more complex targets requiring a longer training paradigm to reach peak performance. Second, the computerized VR test allows the recording of highly detailed testing parameters, such as tracing the movement of the animals during the entire course of the test in high temporal resolution. Third, the computerized VR system runs the test with minimal human-animal interaction during the test (except for the initial training during Acclimation and Target Identification) and, therefore enhances the consistency of the tests and minimizes test variation. Forth, unlike the OKR, which records a reflex-based response to visual stimulation [19–21], the VR visual tests examine the visual perception of the animal. Finally, this VR behavioral test could be combined with in vivo image recording of CNS neurons in awake animals for correlated study of cellular activity of brain and behavior [14,29,30], and therefore, provides a platform to study the cellular mechanisms of visual perception.

There seems to be two major learning components for optimized VR performance. One is to learn the recognition of the correct rewarding visual target, and the other is to learn to navigate through the VR arena. The capability of recognizing visual targets can be evaluated by the ratio of successful arrivals at the designated targets, while the ability of navigating the VR arena can be evaluated by measuring the proportion of distance that mice used to reach the rewarding target, the time required to reach a rewarding target, and the frequency of arriving at the rewarding target. For both the color/luminance targets and moving bars, mice can correctly identify the rewarding targets in a short training period. This is similar to, or less than, the amount of training time required for other behavioral tests, such as the Morris water maze or lever pressing tests [14,23–25,27,29,31,32] but not the reflex-based behavioral tasks, such as OKR [19–21]. However, additional training significantly improved the performance by increasing the frequency of arriving at rewarding targets, and shortening the time required to reach a rewarding target, indicating that the improvement in navigation requires more training than that of target recognition. Therefore, improvement in the maneuverability of the treadmill for navigation could potentially reduce the required training time, and more precisely measure visual perception. In addition, the type of target makes a significant difference in the training requirements, with more visually complex objects requiring more training to reach optimized performance than simple ones.

It is worth pointing out that the four panels in the color/luminance test have a disparity in light intensity in power and, possibly, luminance. Therefore, the recognition and perception of the green panel against other color/luminance panels might not be solely due to spectral recognition per se, but also the difference in perceived luminance. Mice have two opsins that are expressed in cone photoreceptors, a short (S), and middle (M) opsin with peak sensitivity at the 360 nm and 508 nm respectively. These opsins are expressed at different proportions throughout the retina, with M-opsins dominating the ventral retina, and S-opsins are predominant in the dorsal retina, and some cones express both opsins [16,33,34]. This would seemingly make it difficult for them to distinguish color, however it has been shown that with training that mice can detect differences in UV and green, and also color variations across the visual space [33,35]. In addition, it has been suggested that full immersion into a visual environment is required to fully engage rat spatial navigation within the brain and to prevent the animal from thinking of the VR display as a physical object [6,36,37]. However, Gaffen [34] raised the possibility that the VR displays could be used by the animals as either a part of the environment or a large object within the actual environment and it may not matter for certain experimental questions. Several more recent studies demonstrated that a complete “immersion” might not be required for mice to identify and approach a visual target in a VR environment [5,15,30]. Our results also support the notion that mice can recognize visual cues displayed in partially immersive visual environment and use it for spatial guidance. Because none of these previous studies investigated the properties of visual recognition and the training requirement of visual guided VR tests, our study provides useful information to address the questions of how quickly an experimental animal can learn to recognize certain visual cues, how significantly the complexity of visual cues affects the training requirement and performance of animals, and how long the memory of the visual guided task will last once animals are trained.

Visual stimulation based VR behavioral tests have been extensively used for memory evaluation [10–16,30]. Our results confirmed that the visual memory could last for at least 3 weeks after 3 weeks of training. When mice were reintroduced to the VR behavior test after a 22 day “gap,” they were able to achieve a similar level of performance as before the no-training break. In addition, they were able to quickly surpass the performance of the previous training session after additional training. Therefore, not only could the mice maintain some of their skill set before the break, but also the previous experience could facilitate the additional training. This memory capability is particularly useful to evaluate visual function of animals before and after the induction of a disease or treatment.

In conclusion, we have developed a VR test system, which can essay a variety of different aspects of visual perception. Using this system, it is possible to quantitatively characterize the behavioral responses of mice towards virtually any type of visual cues with minimal human-animal interaction to enhance the consistency of the tests and minimize test variation. Our results provide important details regarding the training requirement of different visual cues. If combined with a head-fixed in vivo imaging recording approach, it could provide a platform for correlated in vivo study of neuronal activity in the brain and visual perception.

## Supporting information

S1 Table Detailed results of Fisher's PLSD tests for Fig 5C. (XLSX)

S1 Video Representative performance of mice before and after training. Video recordings of mice performing the VR behavioral task before and after training.
(MP4)
